# Investigating the Effects of the UniReliever® Knee Brace Using Analysis of a Novel Digital Platform, MAI Motion

**DOI:** 10.7759/cureus.106571

**Published:** 2026-04-07

**Authors:** Tanvi Verma, Yan Wen, Paul Lee, Lei Zhang, Xujiong Ye

**Affiliations:** 1 Research, MSK Doctors and Associates, Grantham, GBR; 2 Research, MSK Doctors, Sleaford, GBR; 3 Trauma and Orthopaedics, MSK Doctors and Associates, Grantham, GBR; 4 Computer Science, University of Exeter, Exeter, GBR

**Keywords:** brace, digital, knee alignment, motion, osteoarthritis, sit-to-stand, squat movements

## Abstract

Background: Knee braces are widely used to reduce pain and aid mobility in people living with knee osteoarthritis (KOA). This study aimed to determine whether a widely used knee brace, the UniReliever knee (URK) brace (Thuasne SAS, Levallois-Perret, France), improved knee alignment in patients with KOA using a novel digital platform, MAI Motion (MSK Doctors, Sleaford, United Kingdom).

Methods: The movement of eight patients was recorded using MAI Motion whilst performing three sets of repeated (5x) sit-to-stand (S2S) and squat movements whilst wearing: no brace (control), an inactive brace with no offloading (additional control), or an active brace with offloading (as used in the real world). The URK (TM5+ hinge^2^) brace, a dynamic offloading knee brace, was used, and images were captured using a standard red-green-blue (RGB) camera. Statistical significance was determined by one-way and two-way Analysis of Variance (ANOVA) using GraphPad Prism (Dotmatics, Boston, Massachusetts, United States).

Results: There was a high degree of variability in knee alignment between patients, accounting for over 80% of the variation observed (p<0.0001). The active URK brace significantly improved knee alignment in three patients performing the S2S activity and four patients performing the Squat activity (all p<0.05).

Conclusions: The active URK brace significantly improved knee alignment in up to 50% of patients. The MAI Motion platform provided an objective method to detect subtle changes during movement, making it suitable for the investigation and monitoring of musculoskeletal conditions in remote, web-based settings.

## Introduction

Knee osteoarthritis (KOA) is a chronic, progressive degenerative joint disease associated with joint pain and stiffness, physical disability, and overall functional impairment. It represents one of the most common causes of chronic pain in older adults [1]. It is characterised by the deterioration of articular cartilage, changes in subchondral bone, and alterations in the synovial membrane.

The onset of KOA is determined by the cumulative effects of mechanical stress, which lead to wear and tear of the cartilage in the knee joint over time, a process influenced by factors such as aging, obesity, previous knee injuries, and genetic predisposition [2]. Evidence of the condition is observed in approximately 37% of adults over the age of 60; the clinical impact affects approximately 13% of women and 10% of men [3,4]. The knee joint is more prone to developing osteoarthritis (OA) compared to the hip and ankle due to its weight-bearing function and the effects of rolling-gliding and rotational movements. Eventually, this condition can alter walking patterns and limit the range of motion during movement. In severe cases, the pain from KOA can lead to a significant decrease in quality of life. With the aging population and the increasing prevalence of obesity, KOA has become one of the most common degenerative joint diseases [5,6].

Knee braces are a common conservative treatment option for managing KOA. The category of knee brace required is determined by the type and extent of the knee injury/condition. Static knee braces are designed to provide rigid support and immobilization of the knee and are typically used after surgery or following severe knee ligament injuries [5,7-9]. In contrast, dynamic knee braces are designed to allow controlled movement, providing support whilst permitting flexion and extension. In addition, they redistribute loading across the knee to decrease compressive forces and malalignment of the knee [10-13]. As such, dynamic braces can improve function and stability, reducing pain due to KOA or during post-surgical rehabilitation [10,14].

The dynamic brace used in this study, the UniReliever® knee (URK) (TM5+ hinge2) brace (Thuasne SAS, Levallois-Perret, France), is a unicompartmental brace that employs a three-point leverage system: the thigh shell (anchoring at the femur) and calf shell (anchoring at the tibia) form two points of support, while the crossing straps on the unaffected side (which may be medial or lateral) constitute the third. It is designed for ease of use, featuring self-dosing adjustment via a simple dial to optimise offloading for pain management [15]. In brief, when activated, the crossing straps provide counter pressure to the thigh and calf shells, which offloads stress from the affected compartment, transferring it to the unaffected side. This process reduces compression, inflammation, and pain, enhancing the knee’s function and range of motion by guiding movement and correcting alignment. These improvements can delay the need for surgical interventions in patients with KOA [16].

MAI Motion (MSK Doctors, Sleaford, United Kingdom) is a proprietary markerless motion capture system that employs artificial intelligence and deep learning to provide precise and objective measurement of the kinematics of the joint during dynamic activities [5]. This technology leverages machine learning analysis of three-dimensional (3D) dynamic motion data. At its core is the development, optimisation, and validation of the CRAFT framework, which comprises the evaluation of Control, Repetition, Asymmetry, Flow, and Twist, key parameters essential for understanding functional joint health. The MAI Motion captures detailed biomechanical insights through analysis of markerless motion capture, which provides a real-world, dynamic perspective on joint function and patient movement beyond that afforded by static imaging. It is predicted that this approach will enhance all aspects of the treatment pathway, including earlier diagnosis, more targeted interventions, and streamlined monitoring, helping to personalise treatment strategies.

This study aimed to evaluate the biomechanical effect of the URK brace on knee alignment in patients living with KOA using the MAI Motion platform. Specifically, the study investigated whether activation of the URK dynamic offloading brace altered knee alignment during functional movements. The primary outcome measure was the change in knee abduction-adduction angle during repeated sit-to-stand and squat movement. By comparing motion patterns with no brace, an inactive brace, and an active offloading brace, this study sought to determine whether the URK brace produces measurable biomechanical changes detectable using markerless motion analysis.

## Materials and methods

The study was conducted at MSK Doctors, Lincolnshire, United Kingdom, from June 27, 2023, to July 30, 2024. Each participant provided informed consent for their involvement in the study and for the dissemination of its outcomes. The study was approved by the MSK Doctors Institutional Review Board (approval number: M2300120006).

Study population

Participants were eligible for inclusion if they were adults aged 18 years or older with a clinical diagnosis of knee osteoarthritis affecting either the medial or lateral compartment. All participants were required to have symptoms consistent with compartment-specific knee loading and to be suitable for an offloading knee brace. Participants had to be able to perform sit-to-stand and squat movements independently and to provide written informed consent prior to participation.

Participants were excluded if they had neurological or neuromuscular conditions affecting lower-limb control, or any condition that impaired their ability to safely perform repeated sit-to-stand or squat movements. Additional criteria included intolerance preventing safe application of the knee brace and current participation in another interventional study that could influence knee biomechanics.

Procedure

**Figure 1:**
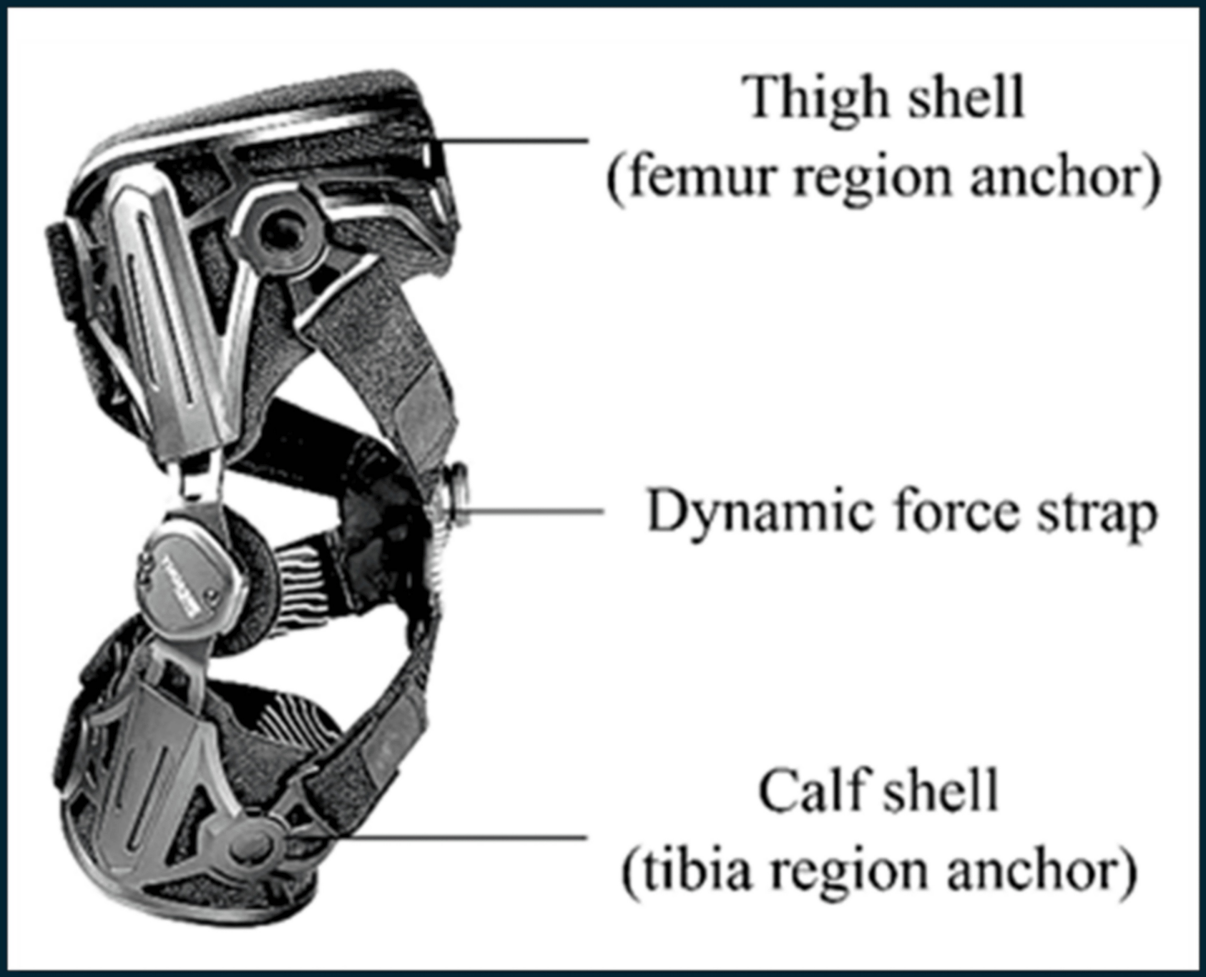
The UniReliever® knee (TM5+ Hinge2) brace. Product Image Source: https://au.thuasne.com/unirelieverr; Figure created by: Authors

The URK brace (TM5+ Hinge2) was used in this study (Figure 1). The appropriate brace was used and fitted in accordance with the manufacturer’s instructions, depending on the affected knee (left or right) and compartment (medial or lateral). Participants were fitted with the brace whilst seated, ensuring the hinge was aligned with the centre of the knee joint. When activated, the dial was engaged to apply the prescribed offloading force to the affected compartment. In the inactive condition, the brace was fitted identically, but the offloading mechanism was not engaged. A standard red-green-blue (RGB) camera (resolution 1920 x 1080, frame rate 30 fps), capable of processing motion tracking and depth sensing, was used to capture movement. The RGB enhances the artificial intelligence (AI) and machine learning capabilities in real-time, making it beneficial for remote monitoring of patients [17,18]. The machine learning model architecture used in this study was the convolutional neural network (CNN) system, MobileNetV2-like, with customised blocks for real-time performance.

MAI Motion Procedure

All recordings were performed in a controlled indoor clinical environment with consistent lighting and a neutral background. Prior to recording, each participant stood facing the camera in a neutral standing position for several seconds while the camera position was adjusted to ensure the participant was visible from head to toe within the frame. Full-body visibility is required for the MAI Motion system to correctly perform motion analysis. If the entire body is not visible, the system is unable to complete the analysis, and an error is generated.

Participants were then instructed to perform the required functional movements. The recorded movements were then processed by the MAI Motion platform to extract joint kinematics.

Data collection and calculations

**Figure 2:**
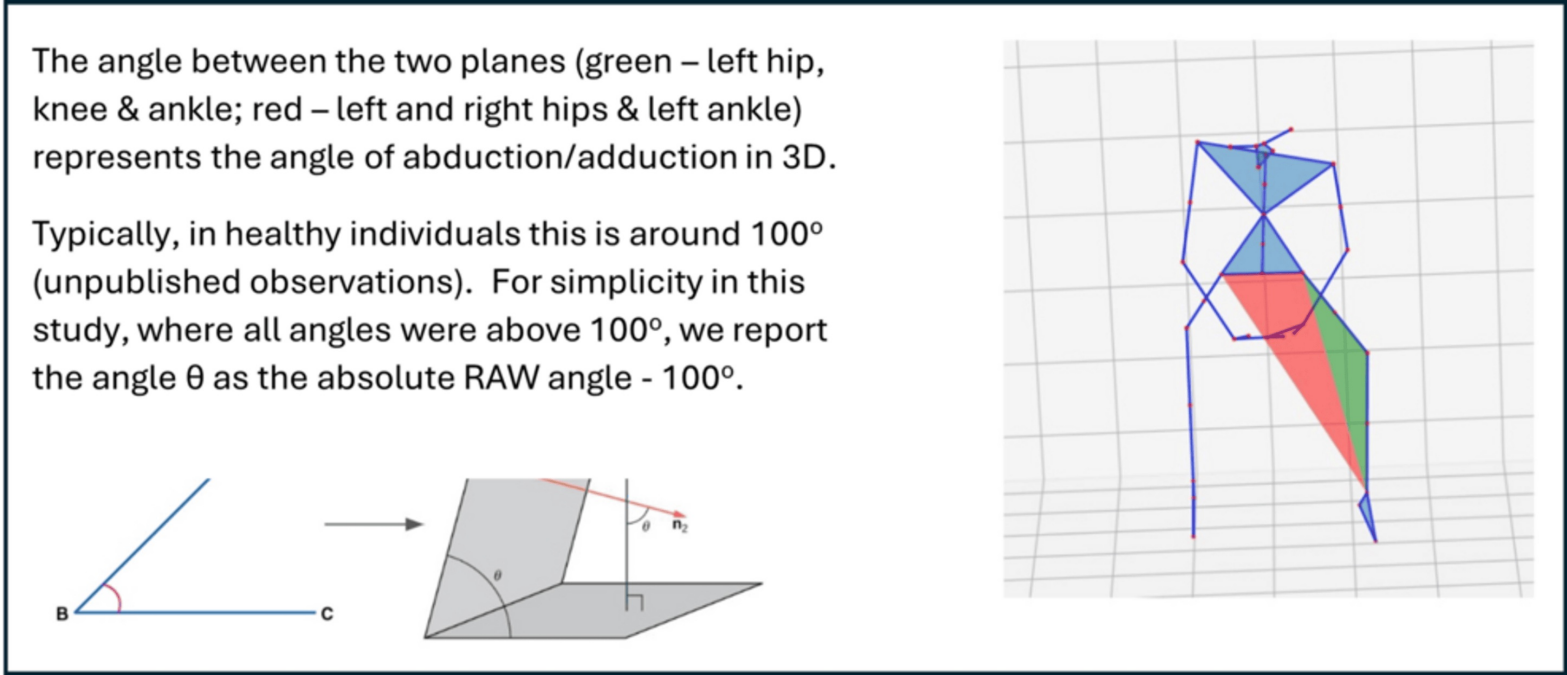
Calculation of the angle of abduction/adduction of the knee in 3D using the MAI motion platform. Image Credit: Authors; created using PowerPoint (Microsoft Corporation, Redmond, Washington, United States) 3D: three dimension

Eight participants were included in the study (seven male and one female; six right and two left knees affected). Each participant was asked to perform three sets of activities. Each set consisted of five sit-to-stand followed by five squat movements (two participants, 1 and 5, were only able to perform three sit-to-stand and three squat activities). Each of the three sets was performed: (i) wearing no brace, (ii) wearing an inactive brace (no offloading) for control purposes, and (iii) wearing an active brace (offloading). The movements of each participant were recorded using a standard RGB camera and processed using the MAI motion platform to calculate the angle of knee abduction/adduction whilst they were performing the activities with different brace statuses (none, inactive, and active). Details of how the angle of abduction/adduction of the knee is calculated in three dimensions (3D) using the MAI Motion platform have been described earlier [5,8] and are summarized in Figure 2. For simplicity, the angle of abduction/adduction (θ) is reported as the RAW angle - 100 ° during each movement. Knee abduction/adduction angle was calculated for each recorded sit-to-stand and squat movement under each brace condition, and these measurements were entered into the statistical analysis.

Statistical analysis

Ordinary one-way or two-way Analysis of Variance (ANOVA) followed by Holm-Šídák’s or Tukey’s multiple comparisons tests were performed using GraphPad Prism version 10.4.1 (Dotmatics, Boston, Massachusetts, United States). Parameters reported included median ± 95% confidence intervals (CIs), mean±standard error of the mean (SEM), and adjusted p values.

## Results

Two-way ANOVA was performed to determine the factors contributing to the variation in knee alignment (defined by the angle θ, which represents the angle of abduction/adduction) in the sit-to-stand (S2S) and squat movements across all eight participants (Table 1). This indicated that variation between participants was the major contributor, representing 96% and 84% in the S2S and squat movements, respectively. Hence, subsequent analyses were performed primarily at the individual participant level, with secondary analyses performed at the group level.

**Table 1:** Sources of variation (N=8) Two-way analysis of variance (ANOVA) was used to analyse data comprising 108 values from eight participants, across three conditions of brace status (None, Inactive, and Active) for both the Sit-to-Stand (S2S) and the Squat movements.

Variblea	S2S		Squat	
	% total variation	p-value	% total variation	p-value
Participants	96.32	<0.0001	84.14	<0.0001
Brace Status	0.2024	<0.0001	2.26	<0.0001
Interaction	2.115	0.0019	10.89	<0.0001

**Figure 3:**
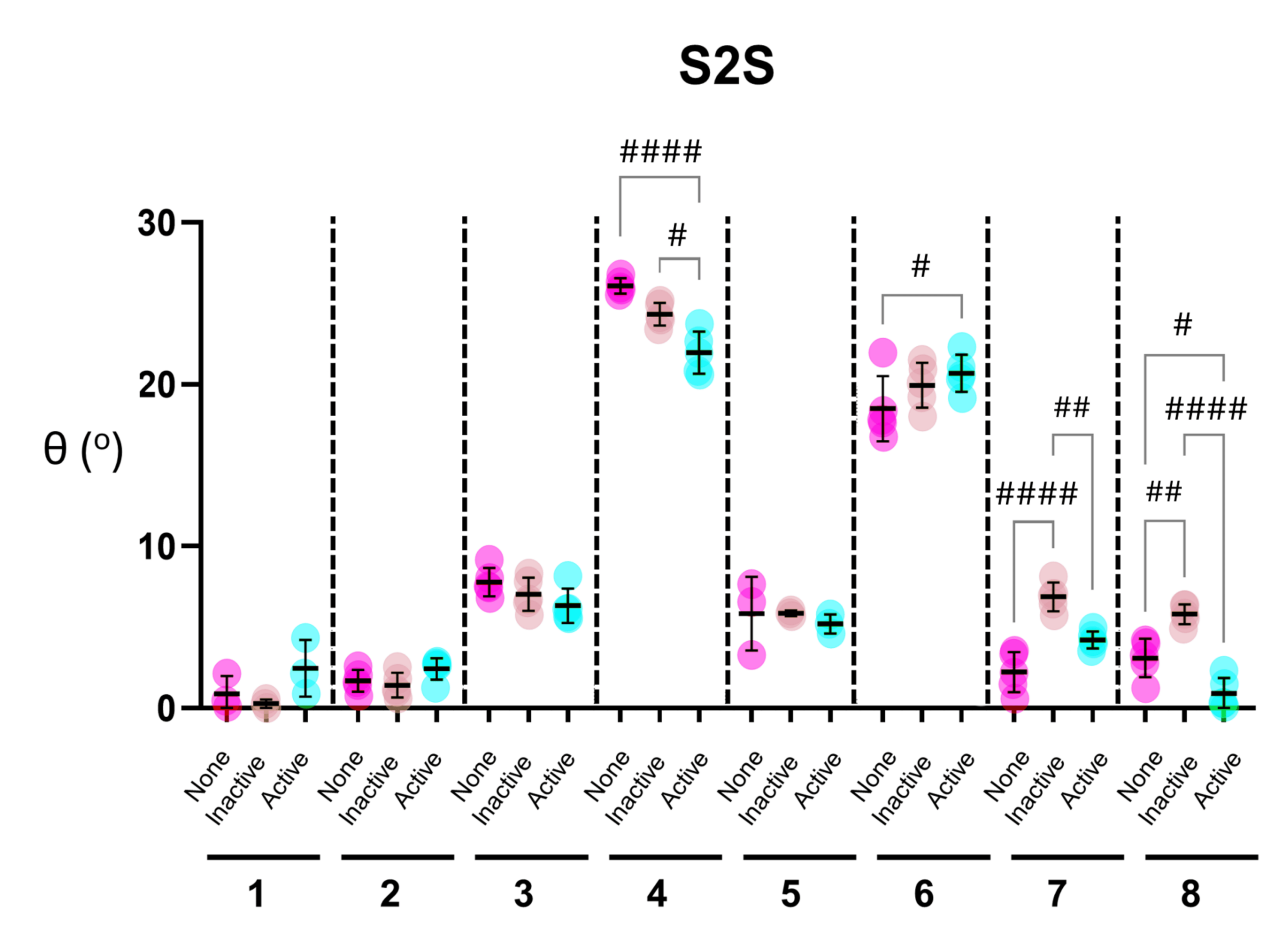
Effect of the UniReliever knee brace on knee alignment in participants performing S2S activities determined by MAI Motion Graphs show angles of knee alignment (θ) from eight participants (numbered 1-8) performing repeated Sit-to-Stand (S2S) activities. All participants performed five repetitions, except participants 1 and 5, who performed three. Individual values and Mean and SD are shown for each participant, with no brace depicted as None (Pink), brace with no offloading as Inactive (tan), and brace with offloading as Active (blue). Statistical analysis was performed using ordinary one-way analysis of variance (ANOVA) followed by Holm-Šídák’s multiple comparisons.  Hash denotes where brace status had a significant effect (#p<0.05, ##p<0.005, ###p=0.0001, ####p<0.0001).

**Figure 4:**
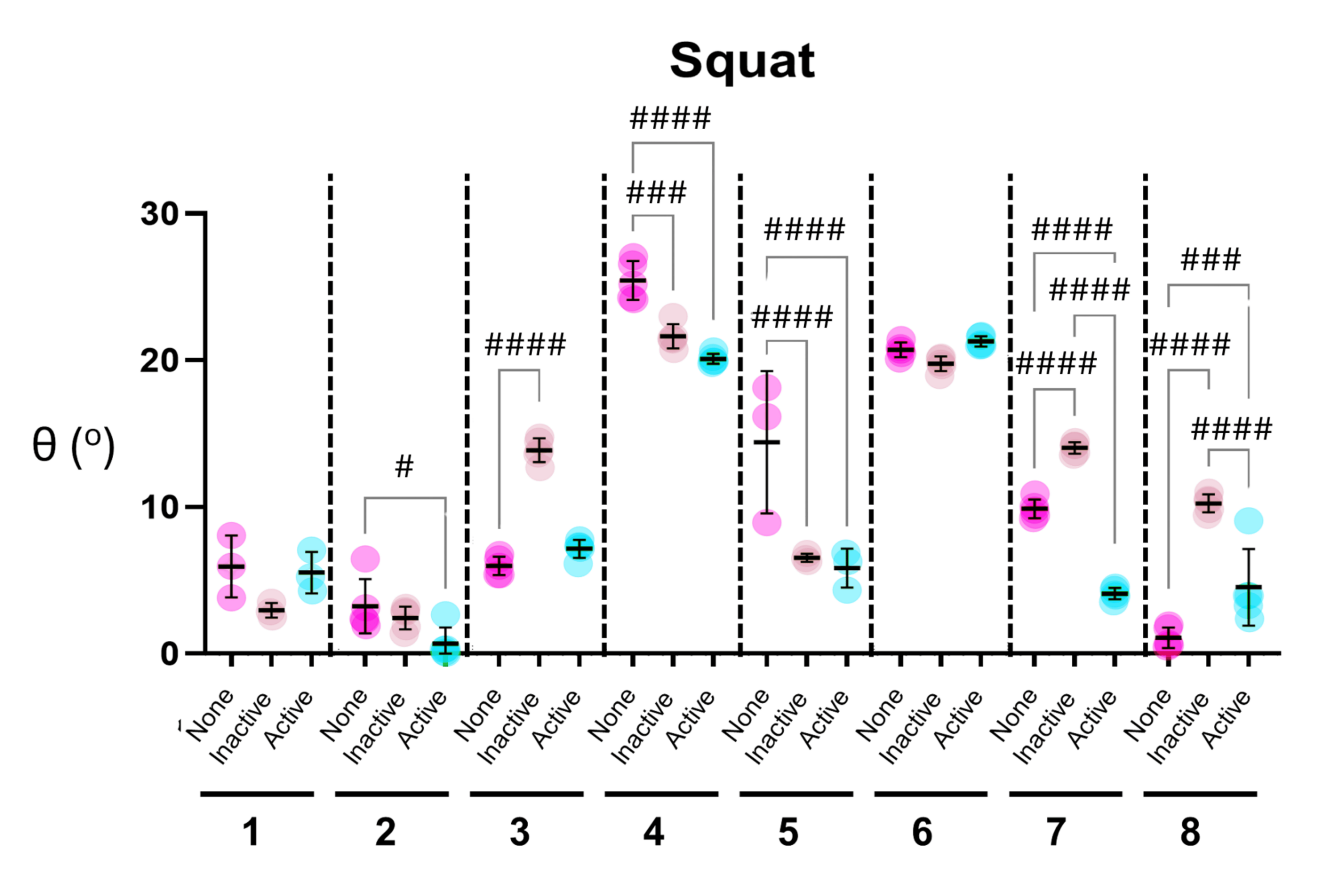
Effect of the UniReliever knee (URK) brace on knee alignment in participants performing Squat activities determined by MAI-Motion. Graphs show angles of knee alignment (θ) from eight participants (numbered 1-8) performing repeated Squat activities. All participants performed five repetitions, except participants 1 and 5, who performed three. Individual values and Mean and SD are shown for each participant, with no brace depicted as None (Pink), brace with no offloading as Inactive (tan), and brace with offloading as Active (blue). Statistical analysis was performed using ordinary one-way analysis of variance (ANOVA) followed by Holm-Šídák’s multiple comparisons. Hash denotes where brace status had a significant effect (#p<0.05, ##p<0.005, ###p=0.0001, ####p<0.0001).

The effect of the URK brace on knee alignment whilst performing the S2S and Squat activities is shown for each participant (Figures 3, 4). Consistent with the initial analysis (see above), the most striking qualitative observation from both datasets was the high degree of variation seen across the participants. Participants 4 and 6 had the highest values, indicative of greater abduction of the knee, when performing both the S2S and Squat movements (Figures 3, 4). Furthermore, there was relatively little variability across repetitions for the S2S or Squat movements within participants, where brace status was constant (either None, Inactive, or Active).

Significant alterations in knee alignment were detected in response to changes in brace status in several participants. For the S2S movement, four of eight participants showed significant changes. Participants 4 and 8 showed significant improvements (θ decreased) when wearing the activated brace (with offloading) (Figure 3). In contrast, participant 6 showed a significant worsening (θ increased) while wearing the activated brace. The effects of the brace on participants 4 and 6 also appeared to be ‘dose-dependent’, with non-significant changes in response to wearing the inactive brace (without offloading) increased upon activation of the brace (Figure 3). The opposite effect was observed in participants 7 and 8, in whom the inactive brace significantly worsened knee alignment, although this effect was significantly reversed upon activation of the brace (Figure 3). 

The effects of the brace were more pronounced in the Squat activity, with six of the eight participants showing significant changes (Figure 4). Four participants, 2, 4, 5, and 7, showed significant improvements when wearing the activated brace, whilst one participant, 8, showed a significant worsening. Perhaps surprisingly, three participants, 3, 7, and 8, showed a significant worsening with the inactive brace, although this was significantly reversed in two participants, 7 and 8, following activation of the brace (Figure 4. These effects were largely consistent across both activities (compare responses in participants 7 and 8 in Figures 3, 4).

The effects of the brace at a group level are summarized in Table 2. The inactive brace had a modest but significant negative impact on knee alignment in both the S2S and Squat movements. Activation of the brace significantly improved knee alignment, reducing knee abduction during the Squat movement and reversing the effect observed with the inactive brace in both S2S and Squat movements.

**Table 2:** Group analysis summary The median ± 95% CIs and mean and standard error of the mean (SEM) of the knee alignment values (θ) are shown for the Sit-to-stand (S2S) and Squat movements. Statistical analysis was performed by two-way analysis of variance (ANOVA) followed by Tukey’s multiple comparisons test. Symbols denote significant differences as follows: *p=0.0291 cf None; ^p=0.0018 cf Inactive; **p<0.0001 cf None; ^^p<0.0001 cf Active in the S2S or Squat movement.

Brace Status	S2S		Squat	
	Median (95% CIs)	Mean (SEM)	Median (95% CIs)	Mean (SEM)
None	4.47 (0.89/26.09)	8.27 (3.25)	7.93 (1.09/25.44)	10.84 (3.06)
Inactive	6.37 (0.28/24.3)	8.95* (3.04)	12.06 (2.44/21.63)	11.43^^ (2.55)
Active	4.71 (0.90/21.96)	8.02^ (2.97)	5.68 (0.29/21.29)	8.65** (2.71)

Collectively, these observations indicate that wearing the URK brace in active mode, with offloading, as would be the case in the real-world, typically elicits a positive effect to improve knee alignment and reduce knee abduction at both an individual and group level. The findings also provide further support for the development and use of the digital MAI Motion platform.

## Discussion

This study aimed to evaluate the impact of the URK brace on alignment of the knee using the markerless MAI Motion capture platform. The findings show that the active URK brace improved knee alignment in some participants, while others showed little change or worsening in patients with KOA when performing routine, everyday movements such as S2S and Squat. These results contribute to the growing body of evidence supporting the use of advanced brace designs for managing KOA [19]. The findings also support further development and use of the MAI Motion platform for objective and remote investigation and monitoring of musculoskeletal conditions.

The active URK brace demonstrated significant improvements in knee alignment, as evidenced by reduced abduction angles during squat movements in four (50%) participants. This indicates that, in these individuals, the offloading mechanism effectively directs the knee toward a more natural coronal alignment. This may reduce the chronic biomechanical stress on the knee joint, as highlighted by the American Academy of Orthopaedic Surgeons [20]. Whilst the benefits of the active brace were less pronounced when participants performed the S2S movements, statistically significant improvements were observed in only two (25%) participants. This may, at least in part, be attributed to the biomechanical differences between squatting and standing motions, where squats involve greater load transfer through the knee joint, potentially amplifying the effect of the brace.

Overall, the findings highlight the potential of the URK brace as a non-invasive supportive intervention for KOA, although responses varied between individuals and improvements were observed only in a subset of participants. Notwithstanding, in those patients where the active brace had limited or even negative effects on knee alignment, refitting of the brace to ensure correct positioning and set-up may improve knee alignment and subsequent biomechanical responses. The integration of MAI Motion, allowing both users and practitioners to measure changes in knee alignment in response to the URK brace or other offloading braces in real time, may facilitate improved fitting and confidence for both patients and practitioners. This approach enables objective assessment of biomechanical responses to brace use and may support more personalised optimisation of brace fitting and use in clinical practice.

Limitations of this study include the small sample size and the limited activities, which restrict extrapolation of the findings and preclude any insights into the long-term impact of the effects of the URK brace on knee alignment and other related outcomes. In contrast, strengths of the study include the use of the markerless MAI Motion platform to provide an objective, reproducible determination of knee biomechanics, allowing repeated evaluations to track brace efficacy [5,8]. Additionally, the use of advanced statistical methods ensured robust analysis of individual and group-level responses.

Future studies should focus on larger, more diverse cohorts to extend the findings and explore the long-term efficacy of the URK brace. Additionally, investigating the brace’s impact on functional outcomes such as pain, mobility, and quality of life would also provide a more comprehensive understanding of its clinical benefits and facilitate economic cost-benefit projections. The integration of patient-reported outcomes with biomechanical data may further refine treatment approaches and improve patient satisfaction.

## Conclusions

This study has shown that the MAI Motion capture system can be used to measure the effects of the URK brace in patients with KOA. Further development and integration of the MAI Motion platform should allow patients and practitioners to optimise fitting by providing objective measurements in real time, affording remote treatment and improved outcomes.
